# Evaluation of the population structure and phylogeography of the Japanese Genji firefly, *Luciola cruciata*, at the nuclear DNA level using RAD-Seq analysis

**DOI:** 10.1038/s41598-020-58324-9

**Published:** 2020-01-30

**Authors:** Dai-ichiro Kato, Hirobumi Suzuki, Atsuhiro Tsuruta, Juri Maeda, Yoshinobu Hayashi, Kazunari Arima, Yuji Ito, Yukio Nagano

**Affiliations:** 1 Department of Chemistry and Bioscience, Graduate School of Science and Engineering, Kagoshima University, 1-21-35 Korimoto, Kagoshima, 890-0065 Japan; 2 Japan Fireflies Society, 2-1-24 Shinmei, Hino, Tokyo, 191-0016 Japan; 3 Department of Biology, Keio University, 4-1-1 Hiyoshi, Kohoku-ku, Yokohama, 223-8521 Japan; 4 Analytical Research Center for Experimental Sciences, Saga University, 1 Honjo-machi, Saga, 840-8502 Japan

**Keywords:** DNA, Genetic variation

## Abstract

The Genji firefly, *Luciola cruciata*, is widely distributed throughout the major Japanese islands (Honshu, Shikoku, and Kyushu) and distinguished into two ecological types on the basis of the flash interval of the mate-seeking males (4-sec slow-flash or 2-sec fast-flash intervals). The boundary of the ecological types corresponds to the Fossa Magna, a great rupture zone that separates eastern and western Japan. Although the degree of genetic differentiation of the two types has been evaluated using allozyme and mitochondrial DNA sequence data, it has not been evaluated using genome-wide data. Based on the genome-wide data obtained using single-end restriction-site-associated DNA (RAD-Seq), principal component, gene-level phylogenetic tree, admixture, and Wright’s fixation index analyses, we identified three phylogenetic groups in *L. cruciata*: East-Honshu, West-Honshu, and Kyushu. This grouping corresponds to the ecological types: East-Honshu to the slow-flash type and West-Honshu and Kyushu to the fast-flash type. Although introgression was exceptionally observed around adjacent or artificially transplanted areas, gene flow among the groups was almost absent in the natural populations. The phylogenetic tree under the coalescent model also evaluated differentiation among the East-Honshu, West-Honshu and Kyushu groups. Furthermore, because the distribution patterns of the three groups are consistent with the geological history of Japanese islands, a vicariant speciation scenario of *L. cruciata* is concluded. In addition, we identified genetic markers that can be used to distinguish the three genetic groups for genetic management of firefly transplantation in nature conservation and regeneration.

## Introduction

The fantastic light of fireflies has been attractive to nearly all peoples. This attraction is particularly strong in Japan, where the firefly has appeared in the oldest Japanese history book, “Nihon Shoki,” compiled during the Nara period of the 8th century.

Light emission in adult fireflies is a mating adaptation^1–3^, and species-specific and mating-related flash signals play a major role in reproductive isolation^4^. Therefore, it is presumed that the flash signal differentiation and speciation processes are closely correlated. The Genji firefly, *Luciola cruciata*, is a species that shows synchronous flashes, and its flash communication and mating behaviour have been intensively studied^5,6^. The communication system is described in several phases: after sunset, the males begin to fly and seek females with synchronous flashing lights; the females emit single-pulsed flashes of light (not synchronized) on grasses; when a male finds the female’s flashed light, the male approaches the female; the male emits flashes with various patterns while approaching and walking around the female; thereafter, they copulate^5–7^.

**Figure 1:**
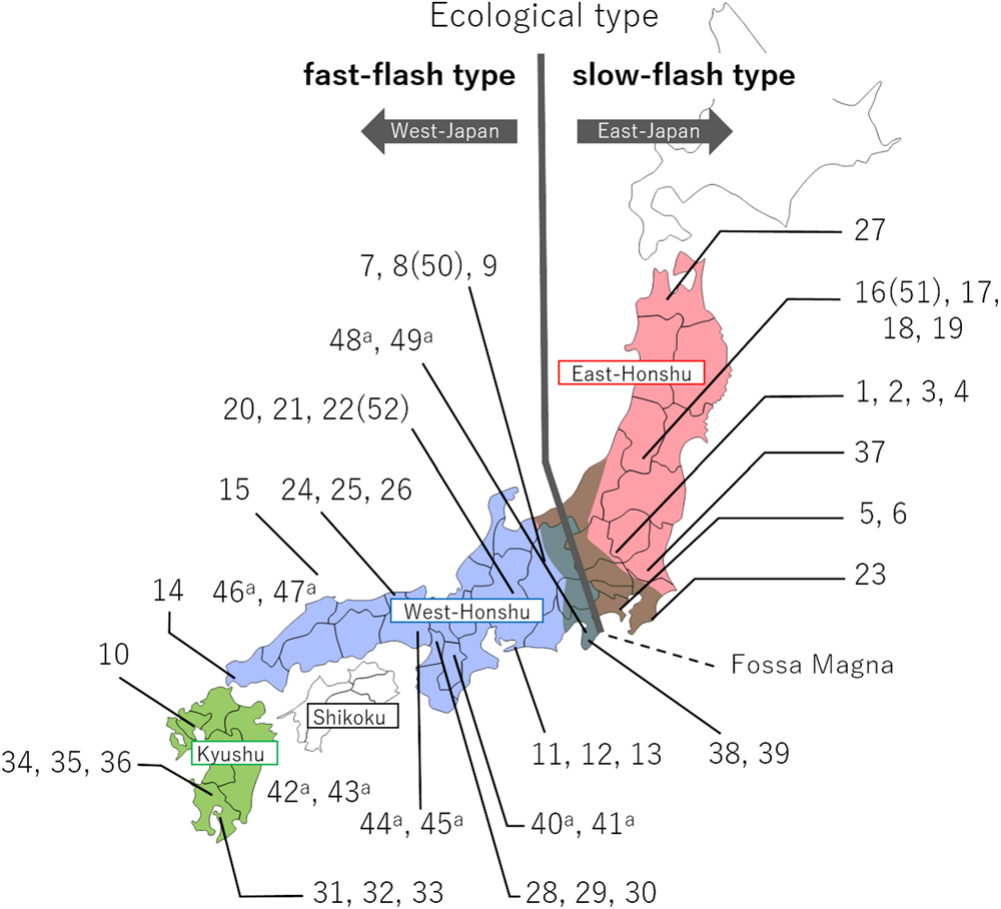
Locality map of *L. cruciata* used in this study. *L. cruciata* is distributed on the three major islands of Japan, i.e., Honshu, Shikoku, and Kyushu. Honshu is divided into eastern and western areas geologically by the great rupture zone, Fossa Magna, shown in grey shading on the map. Collection sites are indicated by the sample (accession) numbers. The superscript letter “a” following the number indicates a purchased sample from distributors, and it can only be identified as an approximate location in Nos. 42, 43, 46, and 47 (Table 1). The sample number in the parentheses is the technical replicate of the previous number (Table 1). The slow-flash type firefly inhabits the eastern area of Honshu (red area), and the fast-flash type the western area of Honshu (blue area) as well as Shikoku and Kyushu (green area).

This species is distributed throughout the three major islands of Japan, i.e., Honshu, Shikoku, and Kyushu, and two ecological types have been recognized based on the synchronous flashing behaviours of males (Fig. 1)^7^. In the slow-flash type, mate-seeking males have a flash interval of approximately 4 sec while in the fast-flash type, they have a flash interval of approximately 2 sec. Furthermore, females of the fast-flash type aggregate at one site for oviposition, whereas those of the slow-flash type do not gregariously assemble. The slow-flash type is distributed in the eastern areas of Japan (East Japan), whereas the fast-flash type firefly occurs in the western areas (West Japan). The habitation boundary corresponds to a great rupture zone termed the Fossa Magna, which divides Honshu island into eastern (East-Honshu) and western (West-Honshu) areas. However, the two types are not morphologically distinguished.

To evaluate the degree of genetic differentiation between the two ecological types, allozyme analysis was performed^8^. The phylogenetic tree showed two major clades. The first was the East-Japan (consistent with the East-Honshu areas) populations, and the other was the West-Japan (including the West-Honshu, Shikoku, and Kyushu areas) populations; these populations corresponded to the slow- and fast-flash types, respectively. The two types were genetically differentiated at the subspecies level (Nei’s genetic distance by allozymes was 0.09)^9^.

Furthermore, the mitochondrial cytochrome oxidase (CO) II gene was also analysed^10^. The phylogenetic tree showed three major clades (I, II, and III). Clade I was composed of haplotypes distributed in East-Honshu, Clade II was in West-Honshu and Shikoku, and the Clade III was in Kyushu. Namely, Clade I corresponded to the slow-flash type, and Clades II and III corresponded to the fast-flash type. Additionally, the phylogenetic tree showed a clade branching; Clade III branched first, followed by branching of Clades II and I. Therefore, the slow-flash type character of Clade I was considered derivative. Moreover, as the branching order nearly corresponded to the formation of the Japanese islands^11,12^, a vicariant scenario concluded that the genetic and ecological diversity in *L. cruciata* would have arisen from phylogenetic separations subsequent to the Japanese island formation process.

In addition to the case of *L. cruciata*, other examples of unique genetic diversity of insects due to division of habitation areas by the formation of the Japanese islands with the Fossa Magna are also known^13–17^. Including them, however, these phylogeographic studies were performed using a small number of genes at the mitochondrial and nuclear DNA level but not using genome-wide DNA data. For genome-wide polymorphism analyses, restriction-site-associated DNA sequencing (RAD-Seq) is a very efficient and cost-effective method^18^. It produces a reduced representation of a genome via high-throughput sequencing for thousands of genomic regions flanked by targeted restriction sites and can provide a huge number of genome-wide polymorphisms across many individuals. The RAD-Seq approach can be used for population genetic studies in wild specimens and in the American firefly^19,20^. In this study, we used genome-wide polymorphism data obtained using the traditional single-end RAD-Seq techniques with the restriction enzyme *Eco*RI, which recognizes a 6-base pair sequence to evaluate the population structure and phylogeography of *L. cruciata* throughout its entire distribution range. Furthermore, we identified genetic markers that can be used to distinguish *L. cruciata* populations.

## Methods

### Sample collection and genomic DNA extraction

Adult *L. cruciata* firefly samples used in this study were 39 individuals collected from 17 localities and 10 individuals purchased by distributors from 5 localities (Table 1); the sample Nos. and localities are mapped in Fig. 1. Most of the samples were males; only two samples, Nos. 26 and 38, were females. Sample Nos. 50, 51, and 52 were technical replicates of Nos. 8, 16, and 22, respectively. The samples were stored in ethanol at room temperature or at −20 °C. Before extraction of genomic DNA, individual samples were air dried for 30 minutes, centrifuged for 1 minute, and air dried for an additional 1 hour to completely remove the ethanol. Then, the entire body excluding the wings was homogenized by freeze grinding using a ball mill. Genomic DNA extraction was performed using the Blood & Cell Culture DNA Mini Kit (Qiagen, Japan) according to the manufacturer’s instructions. The concentration of the isolated genomic DNA was assessed using a QuantiFluor ONE dsDNA System (Promega, Japan).

**Table 1:** *L. cruciata* localities and collection dates used in this study.

Sample No.	Locality	Collecting date	Sample No.	Locality	Collecting date
1	Ashikaga, Tochigi Pref.	June, 1990	27	Hirosaki, Aomori Pref.	July, 1992
2			28	Minoo, Osaka Pref.	June, 2016
3			29		
4			30		
5	Yokohama, Kanagawa Pref.	June, 1990	31	Tarumizu, Kagoshima Pref.	June, 2016
6			32		
7	Matsumoto, Nagano Pref.	July, 1990	33		
8			34	Yusui, Kagoshima Pref.	June, 2016
9			35		
10	Ogi, Saga Pref.	June, 1989	36		
11	Toyohashi, Aichi Pref.	June, 1994	37	Tsukuba, Ibaraki Pref.	June, 2016
12			38	Izu, Shizuoka Pref.	July, 2016
13			39		
14	Toyota, Yamaguchi Pref.	June, 1998	40^a^	Nara Pref. (Yamato-no-kuni)	Purchased June, 2016
15	Nishinoshima, Shimane Pref.	June, 1991	41^a^		
16	Yonezawa, Yamagata Pref.	July, 1989	42^a^	Kyushu area (Yamato-no-kuni)	
17			43^a^		
18			44^a^	Hyogo Pref. (Chikyu)	Purchased June, 2016
19			45^a^		
20	Seki, Gifu Pref.	June, 1997	46^a^	West-Honshu (Riverfashion)	Purchased June, 2016
21			47^a^		
22			48^a^	Shizuoka Pref. (Hotaru-yasan)	Purchased June, 2016
23	Ohara, Chiba Pref.	June, 1990	49^a^		
24	Hamasaka, Hyogo Pref.	June, 1990	50^b^	Same as No. 8	—
25			51^b^	Same as No. 16	—
26			52^b^	Same as No. 22	—

^a^Samples purchased from distributors are within parentheses.

^b^Technical replicates.

### RAD-Seq analysis

The library for RAD-Seq was created according to the established method^21^. *Eco*RI was used as a single restriction enzyme. The library was sequenced by Macrogen (Seoul, South Korea) with 50 bp single-end reads in one lane of an Illumina HiSeq 2000 (Illumina, San Diego, CA, USA). Sequence data were deposited at the DDBJ Sequence Read Archive (DRA) with accession No. DRA007766. Because all firefly samples that were analysed in this study are potentially cross-compatible species, the Hardy–Weinberg principle was considered. The Stacks program is useful for analysing the RAD-Seq data of closely related samples by applying the Hardy–Weinberg principle^22,23^. Therefore, we analysed the RAD-Seq data using the Stacks program. The data were quality filtered using the process_radtags program in the Stacks package (v. 1.46). In addition, adapter sequences and low‐quality ends were trimmed using cutadapt (v. 1.14) and trimmomatic (v. 0.36), respectively^24,25^. The numbers of quality-filtered reads are shown in Supplementary Table S1. The data were aligned with the reference genome of *L. cruciata* (NCBI/DDBJ BioProject number: PRJDB7197) by bowtie2 with the -q–no-unal -L 15 options^26^. The data of the reference genome were used only for information on the nuclear sequences and not used for mitochondrial DNA. Aligned data were analysed using the ref_map.pl script (v. 1.47) of the Stacks package with default parameters.

### Principal component analysis (PCA) and multidimensional scaling (MDS) analysis

The populations program of the Stacks package was used to create a variant call format (VCF) file with the -p 26 option^27^. This option indicates that the minimum number of populations in which a locus must be present to process that locus is 26. In this analysis, because of a reduction in the effect of accidental reads and increased reproducibility, the -p option was set to half of the total 52 samples within the cluster, and locus common to more than one-half of the samples were extracted. The mean sequencing depth for each sample was calculated using vcftools. Principal component analysis (PCA) and multidimensional scaling (MDS) analyses were conducted based on this VCF file using the SNPrelate program (v.1.18.1)^28^.

### Gene-level phylogenetic tree analysis

A multiple alignment was created by aligning the data to the reference genome. The Stacks package populations program was used to create multiple alignments within the cluster using the options -phylip and -phylip_var. The phylogenetic tree based on the maximum likelihood (ML) method was constructed using the RAxML program (version 8.2.12)^29^ (-f = a, -x = 12,345, -p = 12,345, -N (bootstrap value) = 1,000, and -m = GTRGAMMA). Another phylogenetic tree based on Bayesian inference was constructed using the MrBayes program (version 3.2.6)^30^ (lset nst = 6, rates = invgamma, mcmc ngen = 500,000, samplefreq = 1,000, nchains = 4, and savebrlens = yes). In each analysis, the midpoint was used as a root.

### Phylogenetic tree analysis under the coalescent model

Phylogenetic tree analysis was performed using SVDquartets integrated in the software PAUP ver. 4.0a166 using multiple alignments^31–33^. The parameters used for SVDquartets were as follows (Quartet evaluation; Evaluate all possible quartets, Tree inference; Select trees using the QFM quartet assembly, Tree model; Multi-species coalescent; Handling of ambiguities; and Distribute). The number of bootstrap analyses conducted was 1,000 replicates. The same position used in the RAxML and MrBayes analyses was used as a root.

### Admixture analysis

For the admixture analysis, the Stacks population program was used to create a PLINK file with the -p 26 option^34^. The admixture analysis was conducted based on this PLINK file using ADMIXTURE (v. 1.3.0) under default conditions^35^.

### Wright’s fixation indices

To compare the genetic diversity of firefly accessions within and among populations, pairwise F_st_ values were calculated using the Stacks populations program with the –fstats option.

### Genetic marker search and distribution of SNPs

SNPs for grouping *L. cruciata* into two (East- and West-Japan types) or three (East-Honshu, West-Honshu, and Kyushu types) populations in Japan were determined by running two types of our original Ruby scripts. The list of all loci including SNPs was output by the allsnps.rb program using the VCF file. Towards the output file of this calculation, a second program popdiff.rb was run to group the populations. Custom scripts are available on the GitHub web site (https://github.com/yoshinobuhayashi/RADseq_Firefly_2019).

### Mitochondrial CO II gene analysis

The mitochondrial CO II gene was amplified from the genomic DNA using PCR primers (TL2-J-3037: ATGGCAGATTAGTGCAATGG, TK-N-3785: GTTTAAGAGACCAGTACTTG)^36^. Sequence analysis was performed using the Sanger method with BigDye 3.1, and a 3500xL Genetic Analyzer (Applied Biosystems) was used as a DNA sequencer. Sequence data were deposited at BioProject accession No. PRJDB8812. The haplotype tree construction was conducted using MEGA X (ver. 10.0.5)^37^. The nucleotide substitution model best fitted for maximum likelihood (ML) analyses was selected; the ML tree was constructed using the selected parameters. Bootstrap analysis was performed with 1,000 replicates. The closely related species *Luciola owadai* was used as an outgroup.

## Results

### RAD-Seq loci

A comparison of the genome-wide SNPs of the firefly *L. cruciata*, consisting of 52 accessions including 49 individual samples and three technical replicates (Nos. 50–52), was performed. From over 224 million raw single-end reads obtained from single-digest RAD-Seq using the *Eco*RI, 187 million reads were retained after quality filtering with an average of 3.6 million reads per pooled sample (Supplementary Table S1). This number of reads corresponds to 1.12% of the predicted targeted restriction sites on the firefly genome. By catalogue building and SNP calling on reference genome data using Stacks, 435,165 RAD loci that were shared between two or more samples (p-option = 26 of the cut-off value) and 19 loci found in all fifty-two samples (p-option = 52) were obtained. These data were used for population genetics and phylogenetic analyses.

### PCA and MDS analysis

**Figure 2:**
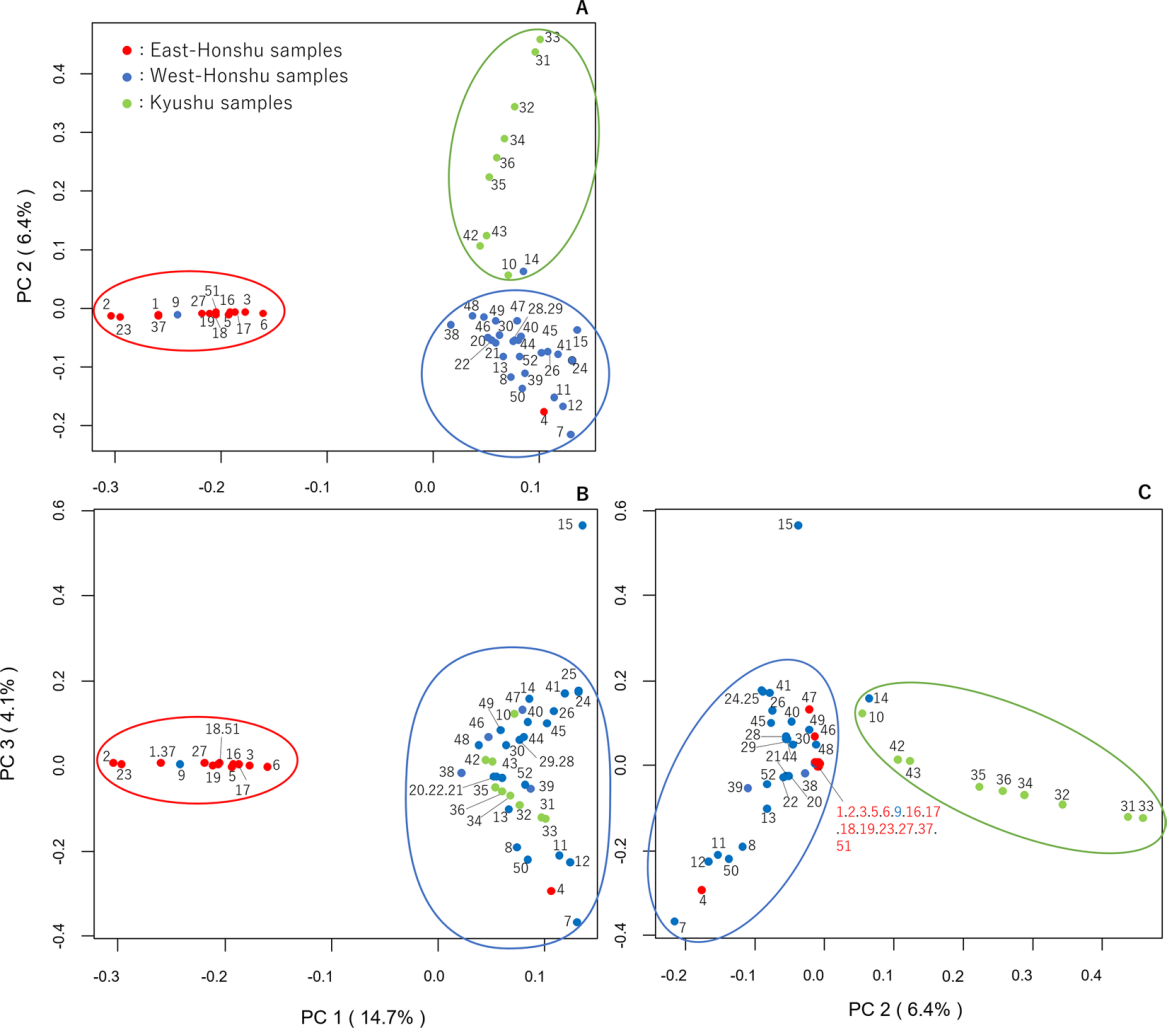
Representation of the *L. cruciata* accessions via principal component analysis (PCA). The three major component data are shown by the three two-dimensional data sets: (**A**) first and second PCA axes, (**B**) first and third PCA axes, and (**C**) second and third PCA axes. The contribution rate of each component is shown in parentheses. The colour of each plotted accession indicates the sample collection area (Fig. 1); the eastern area of Honshu is red, the western area of Honshu is blue, and Kyushu is green. The three clusters are distinguished by the coloured rope hook.

To explore the relationship among the 52 accessions and examine the population structure, PCA was performed; graphs were created using the first three components (Fig. 2). The contribution rate of the three components reached up to 25.2% of the analysed data, broken down as follows: first principle comportment (PC1) = 14.7% of the data, PC2 = 6.4%, and PC3 = 4.1%. Each plot of the accessions was drawn using three colours, i.e., green, blue, and red, based on their collected or purchased area, i.e., Kyushu, West-Honshu, and East-Honshu, respectively. On the graph, firefly accessions were first divided into two groups (the cluster plotted in red vs. the other two colours) on the basis of the values of PC1. The analysis of PC2 indicated a second separation into two other groups (green vs. the other two colours). Consequently, the two-dimensional plots of PC1 vs. PC2 showed a clear separation of three large clusters, and all 52 accessions were clustered into approximately three groups according to the aforementioned habitation areas (Fig. 2A). There were, however, three exceptions in that accession No. 4 (red) was in the blue West-Honshu group, No. 9 (blue) in the red East-Honshu group, and No. 14 (blue) in the green Kyushu group. More detailed information regarding the residential area of each firefly was obtained from the three-dimensional data of MDS analysis; the distribution of the plots of the East-Honshu fireflies (red) tended to be narrower than that of other two clusters and concentrated in a restricted location (Supplementary Fig. S1).

### Gene-level phylogenetic tree analysis

**Figure 3:**
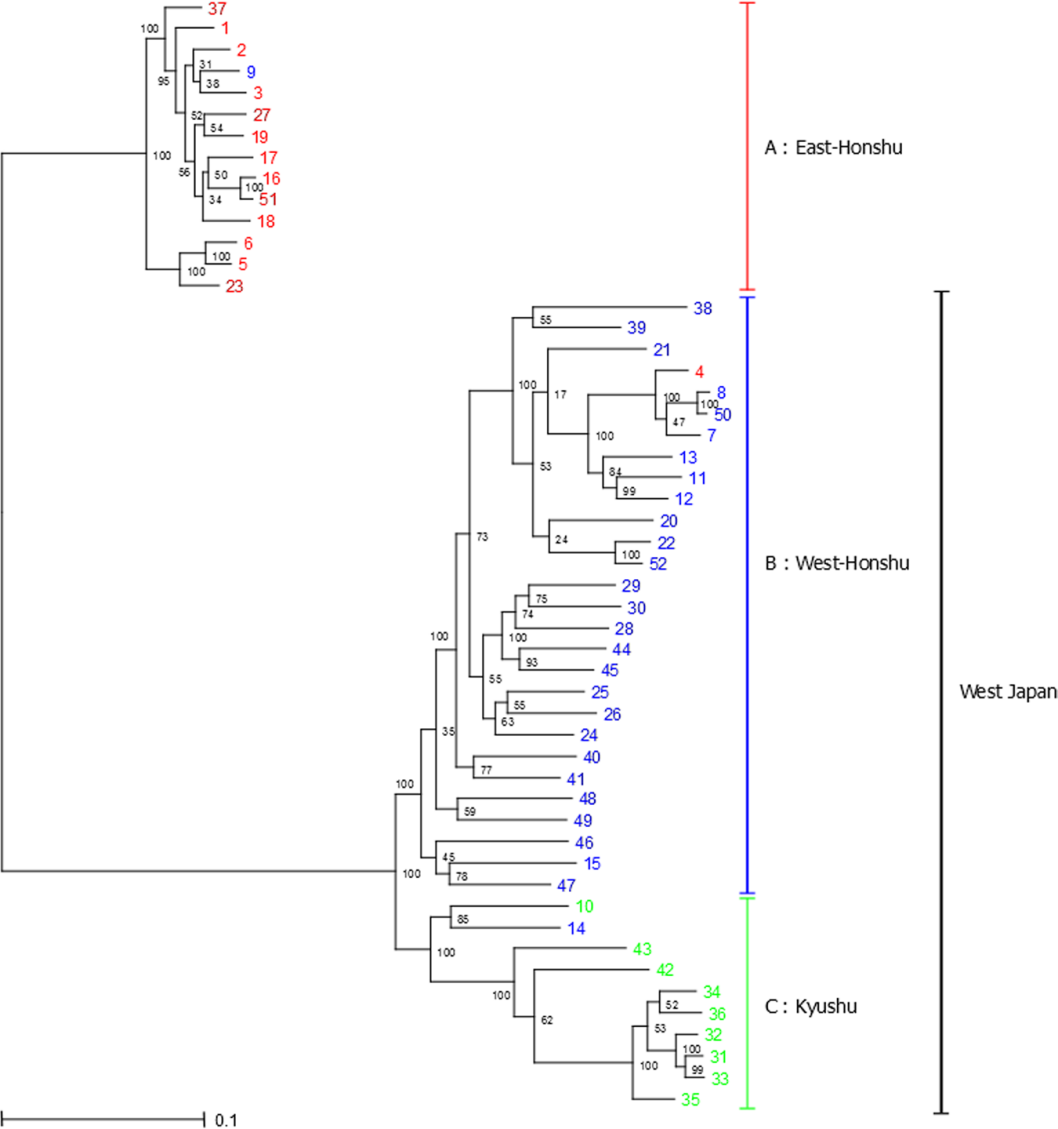
Phylogenetic tree of *L. cruciata* accessions using nuclear DNA data based on maximum likelihood (ML) reconstruction. The numbers at the nodes indicate bootstrap values (% over 1,000 replicates). The scale bar shows the number of substitutions per site. The colours of each accession indicate the sample collection area (Fig. 1); the eastern area of Honshu is red, the western area of Honshu is blue, and Kyushu is green. The right sidebar shows the three major clades divided by the branching pattern of the phylogenetic tree: (**A**) East-Honshu, (**B**) West-Honshu, and (**C**) Kyushu. The midpoint of the longest branch is used as a root.

We constructed two types of gene-level phylogenetic trees at the nuclear DNA level based on the ML method and Bayesian inference analysis (Fig. 3 and Supplementary Fig. S2), in which it was assumed that within-gene recombination was absent. For these analyses, 69,314 phylogenetically informative sites, which were detected by multiple alignments using the reference genome, were used. In addition, the midpoint was set as a root. Notably, this point is estimated as a root using the UPGMA method, in which root estimation is possible. A comparison of the two phylogenetic trees showed no differences in the topology of the trees, and both trees showed two major clades, A and B-C, with the latter clade divided into B and C. The inferred clades were supported by a bootstrap score of 100. The accessions belonging to each clade were consistent with their habitation areas, East-Honshu (Clade A), West-Honshu (Clade B), and Kyushu (Clade C), respectively. This result was the same with the grouping via PCA and MDS analyses with the exception of three accessions; No. 4 collected in East-Honshu was included in Clade B, No. 9 collected in West-Honshu was included in Clade A, and No. 14 collected in West-Honshu was included in Clade C. Based on the PCA results and branch lengths in the nuclear DNA trees, the genome similarity between the West-Honshu and Kyushu fireflies was higher than that between the West- and East-Honshu fireflies.

**Figure 4:**
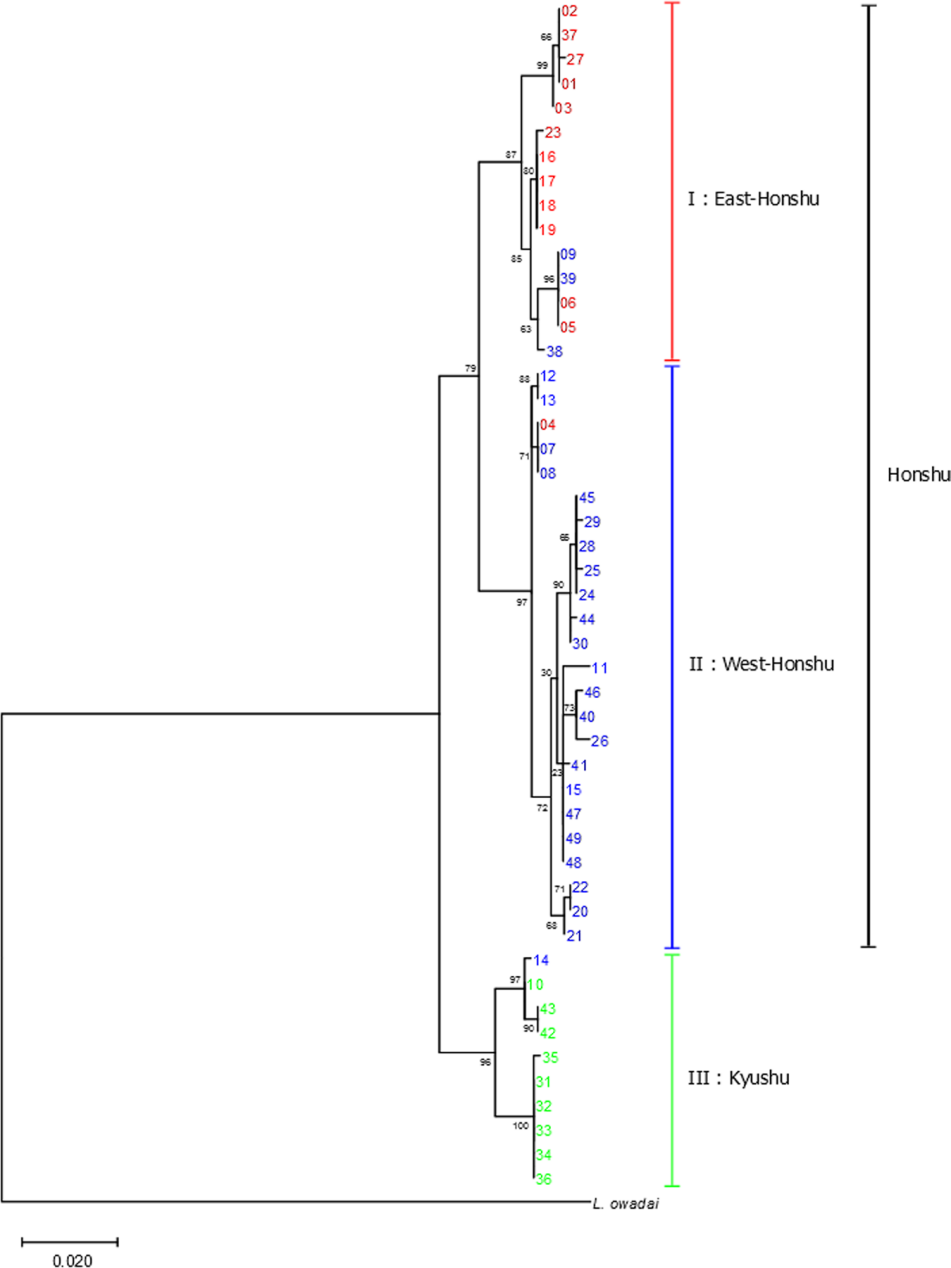
Phylogenetic tree of the haplotypes of *L. cruciata* using the mitochondrial CO II gene based on maximum likelihood (ML) reconstruction. The numbers at the nodes indicate bootstrap values (% over 1,000 replicates). The scale bar shows the number of substitutions per site. The colours of each haplotype indicate the sample collection area (Fig. 1); the eastern area of Honshu is red, the western area of Honshu is blue, and Kyushu is green. The right sidebar shows three major clades: (I) East-Honshu, (II) West-Honshu, and (III) Kyushu. The haplotype of *L. owadai* is used as outgroup.

Another phylogenetic tree using the mitochondrial CO II gene was constructed using the ML method (Fig. 4). The Tamura 3-parameter nucleotide substitution model was selected using the ML method with a discrete Gamma distribution (parameter = 0.1358)^38^. This tree showed two major clades, I-II and III, with the former clade divided into I and II. The haplotypes belonging to each clade were also consistent with their habitation areas: East-Honshu (Clade I), West-Honshu (Clade II), and Kyushu (Clade III). In addition to the same three exceptions of the nuclear tree (Nos. 4, 9, and 14), the haplotypes Nos. 38 and 39 were also exceptional in that they were included in Clade I, although they were collected in the West-Honshu area. The branching order of the clades was different from the nuclear trees, and the mitochondrial genome similarity between the West- and East-Honshu fireflies was higher than that between the West-Honshu and Kyushu fireflies as also found in our previous paper^10^.

### Phylogenetic tree analysis under the coalescent model

**Figure 5:**
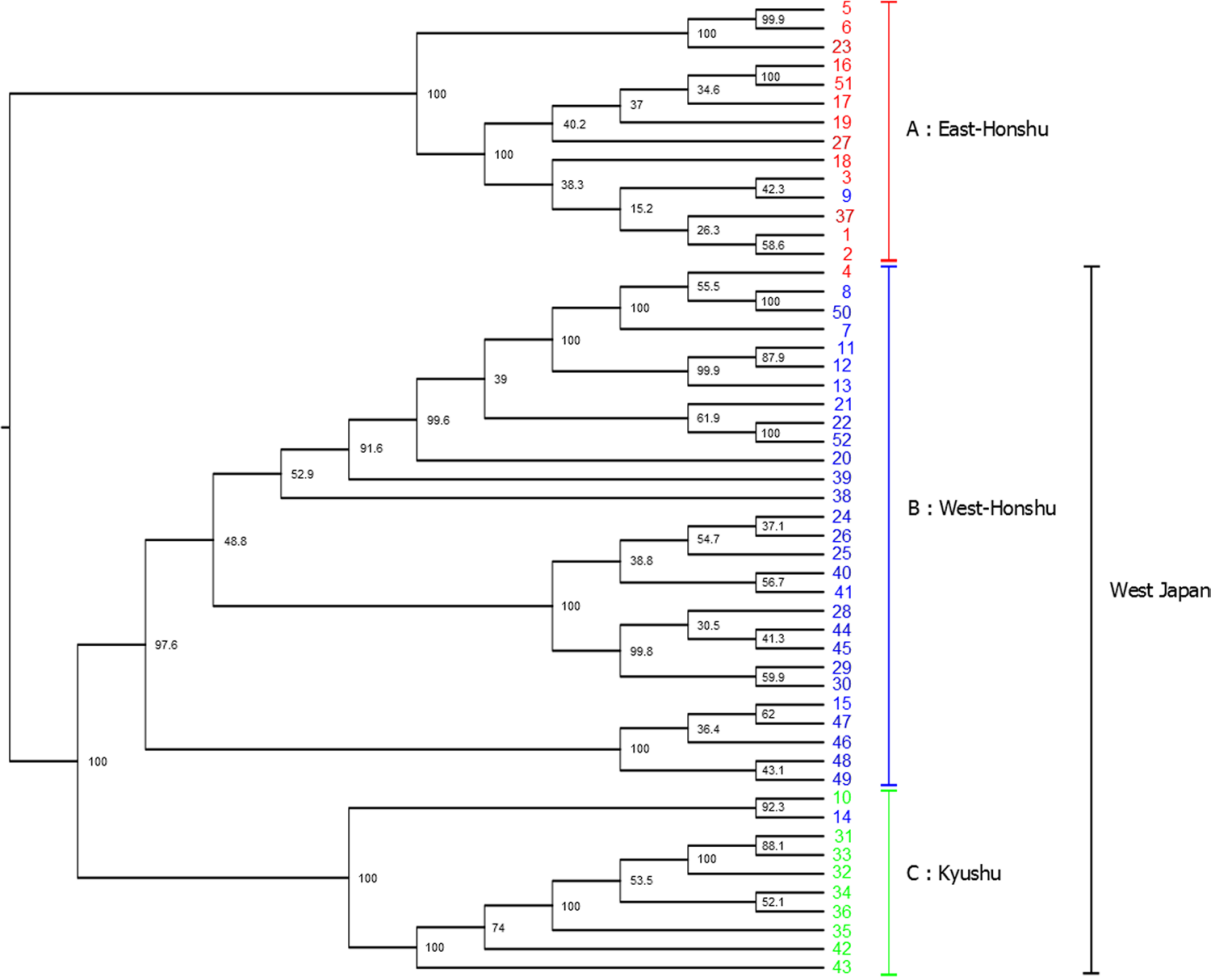
Phylogenetic tree constructed using the SVDquartets method with PAUP using nuclear DNA data of *L. cruciata*. The numbers at the nodes indicate bootstrap values (% over 1,000 replicates). The colours of each accession indicate the sample collection area (Fig. 1): the eastern area of Honshu is red, the western area of Honshu is blue, and Kyushu is green. The right sidebar shows the three groups inferred: (**A**) East-Honshu, (**B**) West-Honshu, and (**C**) Kyushu. The midpoint is used as a root.

In contrast to the aforementioned two methods, which were used to analyse nuclear DNA, in the method under the coalescent model such as SVDquartets, it is assumed that within-gene recombination is present. In the SVDquartets analysis shown in Fig. 5, *L. cruciata* was resolved into two major groups, East-Honshu and West Japan, and the latter group divided into two subgroups, West-Honshu and Kyushu, which is the same as the gene-level phylogenetic trees of ML and Bayesian models (Fig. 3 and Supplementary Fig. S2). The inferred groups were supported by bootstrap scores from 97.6 to 100. The introgression samples (sample No. 9 in the East-Honshu group, No. 4 in West-Honshu, and No. 14 in Kyushu) were also recognized.

### Admixture analysis

**Figure 6:**
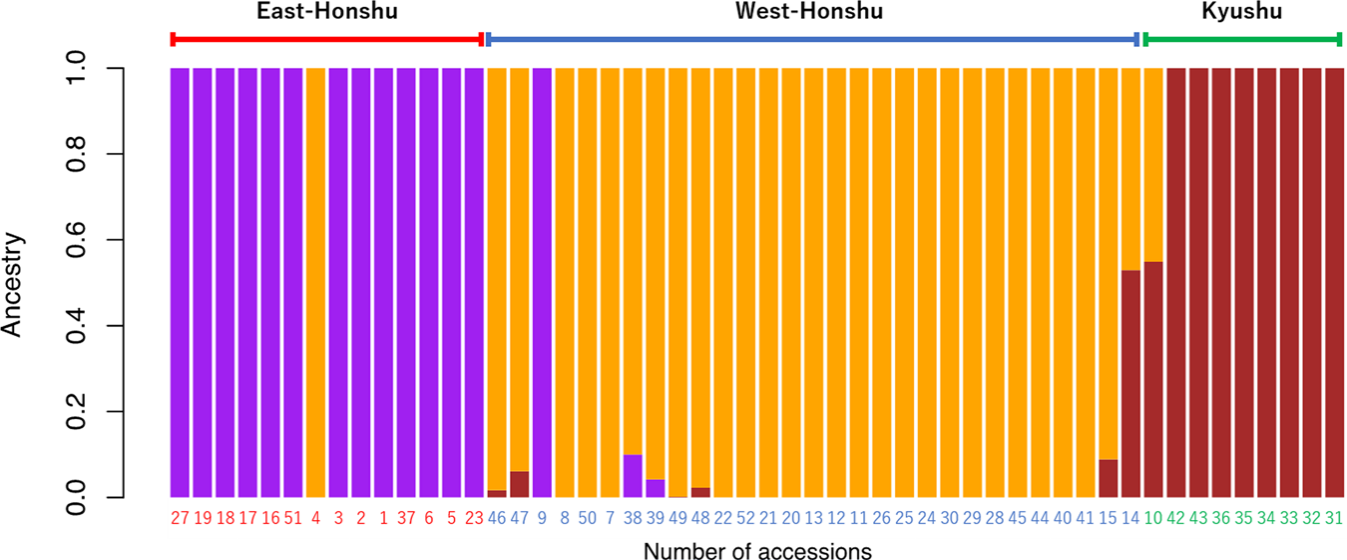
Admixture analysis of the *L. cruciata* accessions. The number of populations (**K**) is set to 3 for admixture analysis. The accessions are horizontally aligned according to the three clades (East-Honshu, West-Honshu, and Kyushu) components of the phylogenetic tree using nuclear DNA data (Fig. 3). The colour of each accession indicates the sample collection area; the eastern area of Honshu is red, the western area of Honshu is blue, and Kyushu is green (Fig. 1). The vertical axis indicates the ancestry ratio of the three hypothetical ancestral populations coloured purple, orange, and brown, respectively.

Based on the results of the admixture program, hypothetical ancestral population (K) value selection was performed from K = 1 to 7. It was found that the error rate was lowest when setting the value as 3 (Fig. 6 and Supplementary Fig. S3). The second lowest error rate was confirmed at K = 2. The sample accessions were horizontally aligned according to their habitation areas, East-Honshu, West-Honshu, and Kyushu; the vertical axis indicated the ancestral ratio of the three hypothetical ancestral populations, which were coloured in purple, orange, and brown, respectively. The distribution of the three hypothetical ancestral populations was consistent with the habitation area of the accessions, which was the same as the grouping via PCA and MDS analyses (Fig. 2 and Supplementary Fig. S1). The clustering via nuclear and mitochondrial DNA trees (Figs. 3 and 4 and Supplementary Fig. S2) had the same exceptions of accessions Nos. 4, 9, and 14. However, no gene mixing was observed in the No. 4 and 9 fireflies, though it was observed between the West-Honshu and Kyushu areas in the No. 10, 14, 15, 46, 47, and 48 accessions. The No. 10, 14, and 15 fireflies inhabited the border area between the West-Honshu and Kyushu areas; however, the original habitation area of Nos. 46 and 47 were not identified because they were purchased by distributors. Gene mixing between the East- and West-Honshu areas was also observed in Nos. 38 and 39; >90% of the genome was from a hypothetical population of West-Honshu, although their mitochondrial haplotypes were confirmed as the East-Honshu type (Fig. 4).

### Wright’s fixation indices

According to the result of admixture and phylogenetic tree analyses, the firefly accessions in this study can be reasonably divided into 3 groups; East-Honshu, West-Honshu, and Kyushu fireflies. To estimate the degree of genetic diversity among and within the three groups, 2 types of pairwise F_st_ values were calculated.

First F_st_ is the value among the three groups, and was calculated as 0.347794 (East-Honshu vs. West-Honshu), 0.546587 (East-Honshu vs. Kyushu), and 0.199462 (West-Honshu vs. Kyushu), respectively (Table 2). The lowest value was observed in the West-Honshu vs. Kyushu firefly sets, and the highest value in the East-Honshu vs. Kyushu sets. These results indicate that the genetically closest pair is the West-Honshu and Kyushu groups and that the East-Honshu and Kyushu groups are the furthest. This result was the same as that of the PCA, MDS, and phylogenetic tree analyses using nuclear DNA but different from that of the mitochondrial DNA analysis.

**Table 2:** Pairwise F_st_ values among the three groups of the phylogenetic tree using nuclear DNA data (Fig. 3): East-Honshu, West-Honshu, and Kyushu in *L. cruciata*.

	East-Honshu	West-Honshu	Kyushu
East-Honshu		0.347794	0.546587
West-Honshu			0.199462

**Figure 7:**
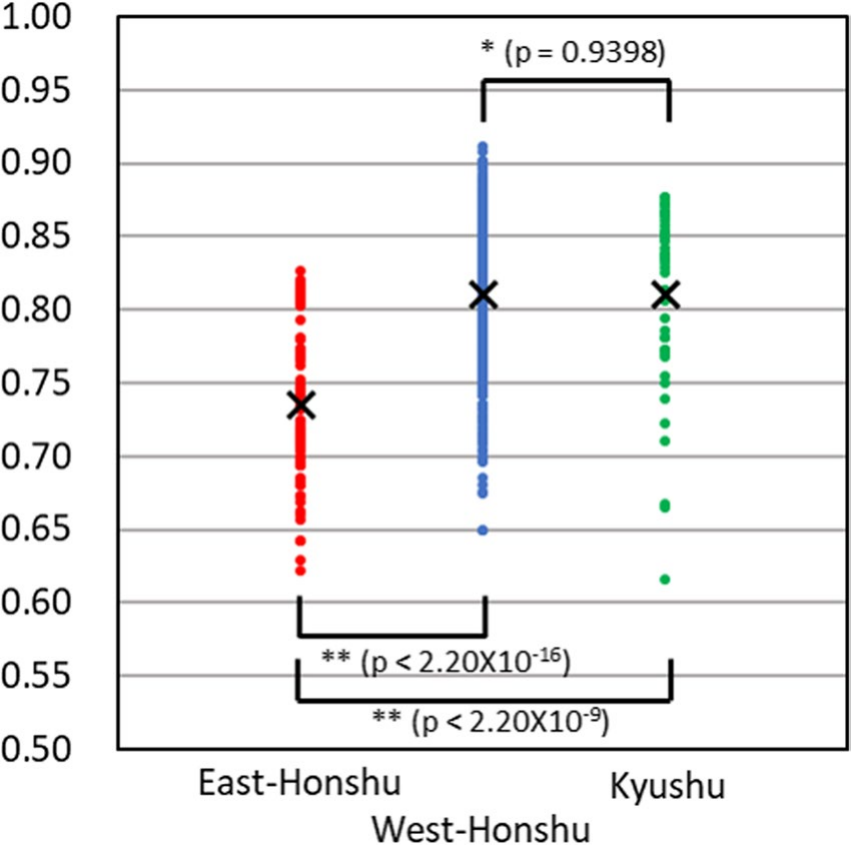
Plot of pairwise F_st_ values among accessions within the three groups of the phylogenetic tree using nuclear DNA data (Fig. 3): East-Honshu, West-Honshu, and Kyushu clades. Details of each F_st_ value are shown in Supplementary Table S2. The averaged value for each group is indicated by a cross mark. *: no significant difference, **: significantly different at the 5% level (p < 2.20 × 10^−16^ between East- and West-Honshu; p < 2.20 × 10^−9^ between East-Honshu and Kyushu).

Second F_st_ is the value among accessions within each group (Supplementary Table S2). These pairwise F_st_ values were plotted in Fig. 7 and average values were calculated as 0.73559 (in East-Honshu), 0.81016 (in West-Honshu), and 0.81076 (in Kyushu), respectively. The East-Honshu group significantly had lowest genetic diversity of the groups (p < 2.20 × 10^−16^ between the East- and West-Honshu groups; p < 2.20 × 10^−9^ between the East-Honshu and Kyushu groups),while the average value between the West-Honshu and Kyushu groups was not significantly different (p = 0.9398).

### Genetic marker search and distribution of SNPs

**Figure 8:**
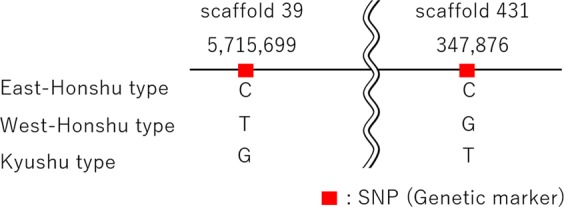
Genetic markers to distinguish the three *L. cruciata* groups (East-Honshu, West-Honshu, and Kyushu). The two SNP sites that can distinguish the three groups are identified in the noncoding gene region of the *L. cruciata* genome.

To distinguish the three groups, genetic markers were searched and two SNP sites were identified (Fig. 8). These two SNPs were positioned in the noncoding region of the *L. cruciata* genome. In addition, other genetic markers were also searched to divide them into East-Japan (consistent with East-Honshu) and West-Japan (including West-Honshu, Shikoku, and Kyushu) groups; 2,545 SNPs were predicted on the entire genome sequence of *L. cruciata*. The results are shown in Table 3 and Supplementary Table S3. Although 1,964 SNPs were positioned in the noncoding region, the remaining 581 SNPs were located in the gene regions. Among them, 215 SNPs were distributed on protein-coding sequences (CDS), and in particular, 77 SNPs were found to specifically cause amino acid substitutions at translated protein levels.

**Table 3:** SNP sites that distinguish the West- and East-Japan types on the entire *L. cruciata* genome.

Region	Gene region			Noncoding region	Total
	CDS	UTR	Intron		
Number of SNPs	215	98	268	1,964	2,545

## Discussion

The results of PCA, MDS, gene-level phylogenetic tree, and admixture analyses using nuclear DNA data show that 52 accessions of *L. cruciata* are separated into three groups, East-Honshu, West-Honshu, and Kyushu types (Figs. 2, 3, and 6 and Supplementary Fig. S1). The grouping is also confirmed by the phylogenetic tree, in which the within-gene recombination is considered (Fig. 5). The groups geologically and geographically correspond to the eastern and western areas of Honshu divided by Fossa Magna and Kyushu. Gene flow among the groups is almost absent in natural populations. Exceptionally, accession Nos. 4 and 9 were collected in East- and West-Honshu, but were unequivocally placed in the West- and East-Honshu group, respectively, according to both their nuclear and mitochondrial DNA. Therefore, it is speculated that the two firefly specimens were artificially transplanted to the collection areas. Meanwhile, accession No. 14 collected from West-Honshu is characterized in the Kyushu group. The collection site is geographically very near Kyushu, and 65% of the nuclear DNA belongs to the Kyushu group. Therefore, it is conjected that the firefly specimen is a progeny of the crossing between West-Honshu and Kyushu fireflies. However, it is unknown whether the sample originates from artificial transplantation or natural migration. Although nuclear DNA introgression among the groups is rarely observed, there is a possibility of intercrossing in a close area naturally or by artificially transplanted specimens in the seven accessions Nos. 10, 15, 38, 39, 46, 47, and 48 (Figs. 3 and 6 and Supplementary Fig. S2). Remarkably, the Izu population (accession Nos. 38 and 39) is included in the West-Honshu group at a nuclear DNA level but in the East-Honshu type at the mitochondrial DNA level (Clade I in Fig. 4). This result means there is a possibility of backcrossing for paternal characteristics in the first filial generation (F1) between females from East-Honshu and males from West-Honshu^39^. The tree under the coalescent model shows two major groups, East-Honshu and West Japan, and the latter is divided into West-Honshu and Kyushu subgroups (Fig. 5). Because the East-Honshu and West Japan groups correspond to the ecological types, the slow- and fast-flash types, respectively, the major two groups are differentiated by the coalescent model analysis. Ecologically, it is recognized by our field observations that not only the flash interval of mate-seeking males but also their flying speed and activity hours and the gregarious oviposition behaviour of females were differentiated between the eastern and western areas of Japan^7,40^. Furthermore, preferential swarming activity of males for artificial flash light was also differentiated in that the males of the slow-flash population preferentially responded to a longer flash interval (4 and 5 sec),while those of the fast-flash type population to a shorter flash interval (2 and 4 sec); pre-mating isolation was suggested^41^. Chromosomal polymorphism is also observed in two populations in that the number of chromosomes in the East-Honshu (Kanagawa prefecture) and West-Japan (Ehime prefecture, Shikoku) is 2n = 20 and 18, respectively^42,43^. Further studies are necessary to clarify the chromosomal differentiation and fertility between the two chromosomal types. At least considering that the gene flow among the three groups is almost absent in natural populations and the grouping corresponds to the two ecological types (flash signal differentiation), *L. cruciata* appears to be in the process of speciation in the Japanese islands.

The consistency between the distribution pattern of the three groups and the geological structure of the Japanese islands implies a vicariant speciation scenario for this species. According to the geological history of Japanese island formation, the ancient landmass of the island was separated from the eastern periphery of the Eurasian Continent and was divided into east and west landmasses during the early phase of Fossa Magna formation approximately 16 million years (MY) ago^11,12^. A considerable part of the current eastern area of the Japanese islands was island-like, mostly under the sea and formed by subsequent crustal uplift (13 MY ago); an outline of the current Japanese islands (Hokkaido, Honshu, Shikoku and Kyusyu) was established from 0.1 to 0.5 MY ago. All trees via nuclear DNA analysis show the branching order of the three groups: the East-Honshu and West-Honshu–Kyushu branch first, followed by branching of West-Honshu and Kyushu (Figs. 3 and 5 and Supplementary Fig. S2). Thus, it seems more likely that the split occurred first between the populations of East and West Japan, and later between West-Honshu and Kyushu. The East–West first scenario is also supported by the PCA and MDS (Fig. 2 and Supplementary Fig. S1), the branch lengths (which lead to the midpoint root) of the nuclear ML tree (Fig. 3 and Supplementary Fig. S2) and the K = 2 admixture analysis (Supplementary Fig. S3). Furthermore, because the F_st_ values among the accession within each group were significantly lowest in the East Honshu population, it may have undergone a bottleneck effect due to island formation via subsidence and uplift (Fig. 7 and Supplementary Table S2). Thus, in conclusion, we argue the East-West divergence happening first is more likely and better supported by the data. On the other hand, the absence of a nuclear DNA outgroup means the branching order cannot be accurately reconstructed. The rooted mitochondrial tree even suggests a different branching order, which could hint at a different evolutionary history for the mitochondrial DNA (Fig. 4). Both vicariance scenarios (East–West or Honshu–Kyushu first) have been reported from other insects^13^. The evolutionary process of the ecological types is not parsimoniously conclusive, because both scenarios, slow- or fast flash type are ancestral, require the same number of evolutionary steps. In addition, it is ambiguous whether the flash type of *L. owadai*, a closely related species of *L. cruciata* and used as an outgroup in mitochondrial DNA tree preparation, is an Eastern slow- or a Western fast flash type.

Finally, SNP markers are presented in Fig. 8, Table 3, and Supplementary Table S3. *L. cruciata* is a popular firefly in Japan and is used as a symbolic insect for nature conservation and regeneration. In this effort, artificial transplantation among the three groups was observed via mitochondrial CO II gene analysis^44^. The SNP markers of the nuclear DNA would also be available for genetic management of firefly transplantation in nature conservation and regeneration.

## Supplementary information

Supporting Information.
