# Supplementary material for: Evaluation of the population structure and phylogeography of the Japanese Genji firefly, Luciola cruciata, at the nuclear DNA level using RAD-Seq analysis
Source: Sci Rep. 2020 Jan 30;10:1533. doi: 10.1038/s41598-020-58324-9 (PMC6992745; doi:10.1038/s41598-020-58324-9)
Supplement: Supplementary file 1 — Supporting Information. [file 41598_2020_58324_MOESM1_ESM.pdf]

Supplementary Materials

Evaluation of the population structure and phylogeography of the Japanese Genji firefly, *Luciola cruciata*, at the nuclear DNA level using RAD-Seq analysis.

Dai-ichiro Kato*[1], Hirobumi Suzuki[2], Atsuhiro Tsuruta[1], Juri Maeda[1], Yoshinobu Hayashi[3], Kazunari Arima[1], Yuji Ito[1], Yukio Nagano[4]

1) Department of Chemistry and Bioscience, Graduate School of Science and Engineering, Kagoshima University, 1-21-35 Korimoto, Kagoshima 890-0065, Japan
2) Japan Fireflies Society, 2-1-24 Shinmei, Hino, Tokyo 191-0016, Japan
3) Department of Biology, Keio University, 4-1-1 Hiyoshi, Kohoku-ku, Yokohama 223-8521, Japan
4) Analytical Research Center for Experimental Sciences, Saga University, 1 Honjo-machi, Saga 840-8502, Japan

*Corresponding author (E-mail: kato@sci.kagoshima-u.ac.jp)

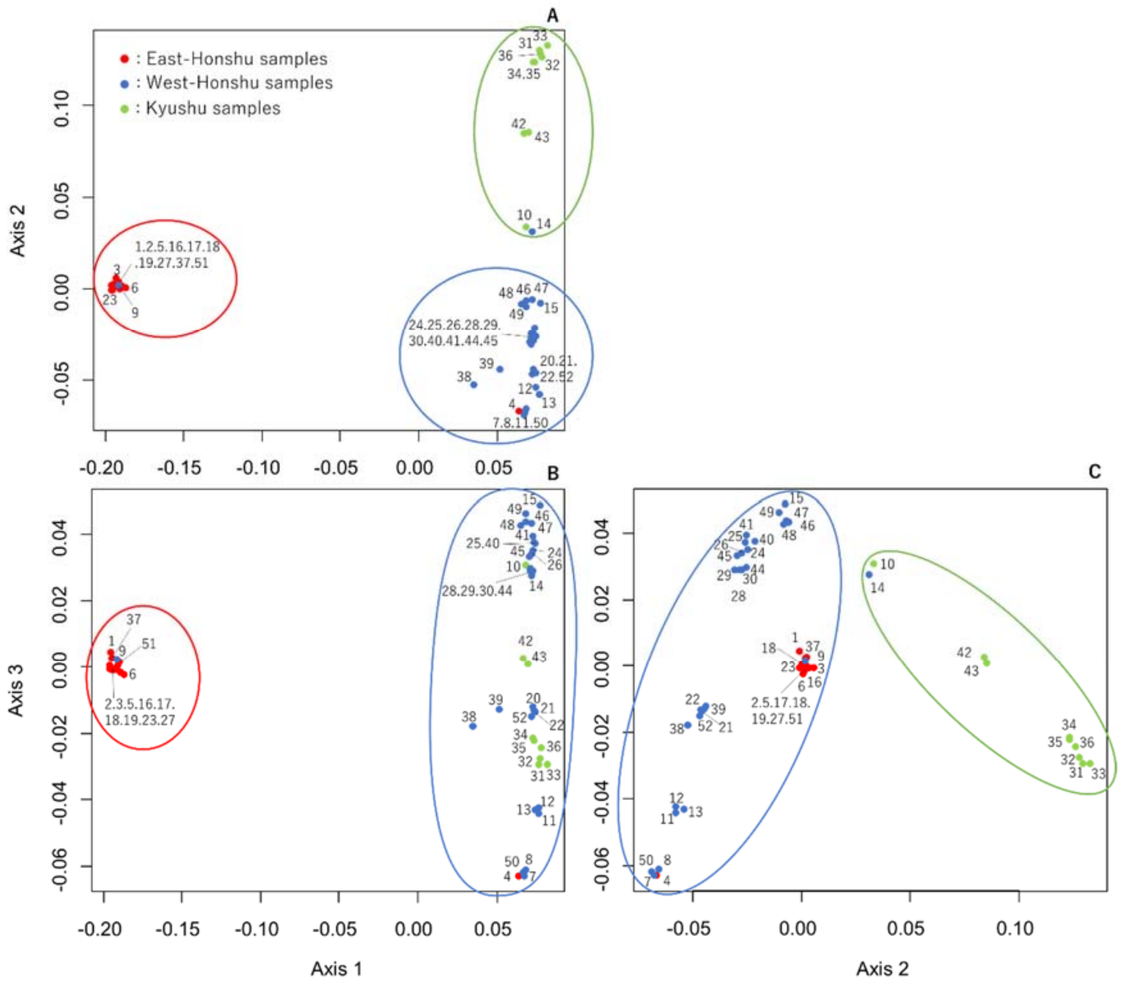

Supplementary Fig. S1. Multidimensional scaling (MDS) representation of the accessions used in this study. Three-dimensional data were obtained in this analysis. Three-dimensional data are shown by three two-dimensional data sets. (A) Axis 1 and 2. (B) Axis 1 and 3. (C) Axis 2 and 3. The colors of plotted each accessions indicate the sample collected or purchased area; East-Honshu as red, West-Honshu as blue, and Kyushu as green, respectively. Colors of rope hook were used to distinguish between three clusters.

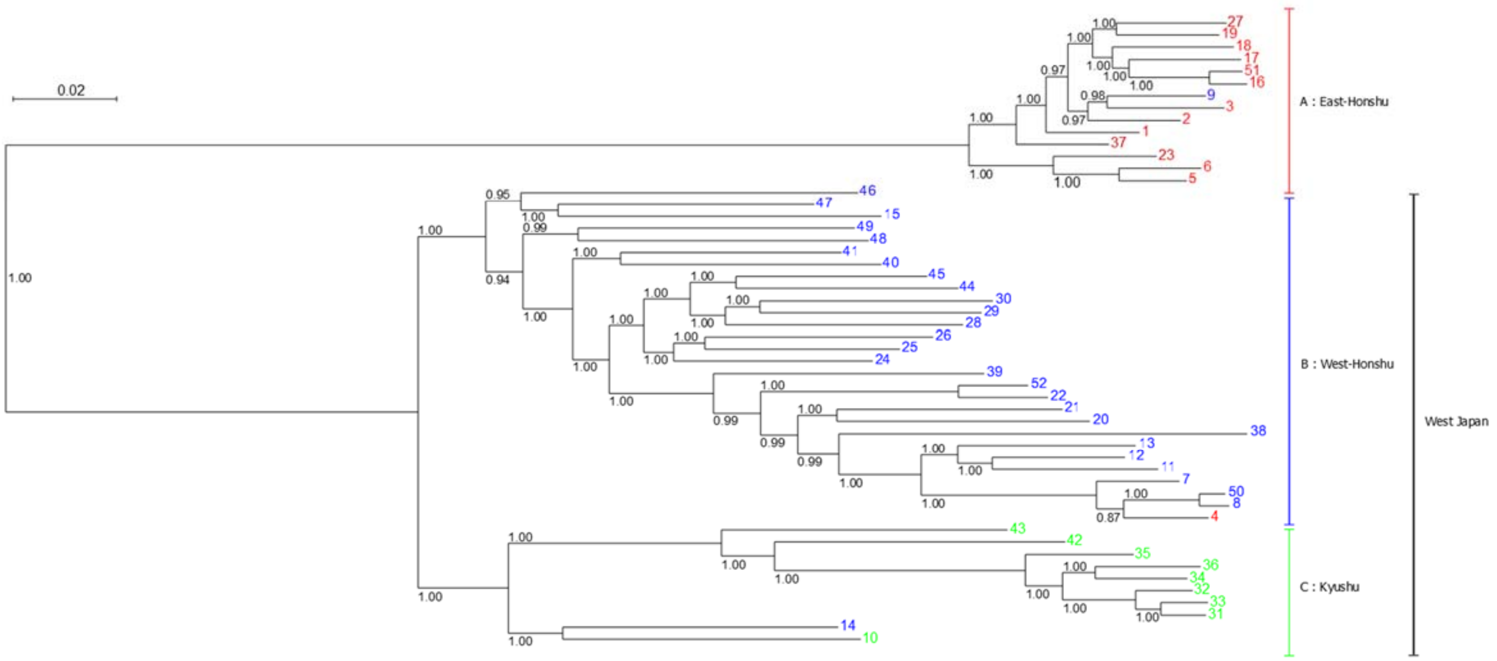

Supplementary Fig. S2. Phylogenetic tree based on Bayesian inference analysis using genome-wide data of *L. cruciata*. Numbers at the nodes indicate posterior probabilities. The scale bar shows the number of substitutions per site. The colors of accession show the collected or purchased area of firefly samples; East-Honshu as red, West-Honshu as blue, and Kyushu as green. The right side bar shows three major clades divided by branching pattern of phylogenetic tree; A: East-Honshu as red, B: West-Honshu as blue, and C: Kyushu as green. Midpoint of the longest branch is used as a root.

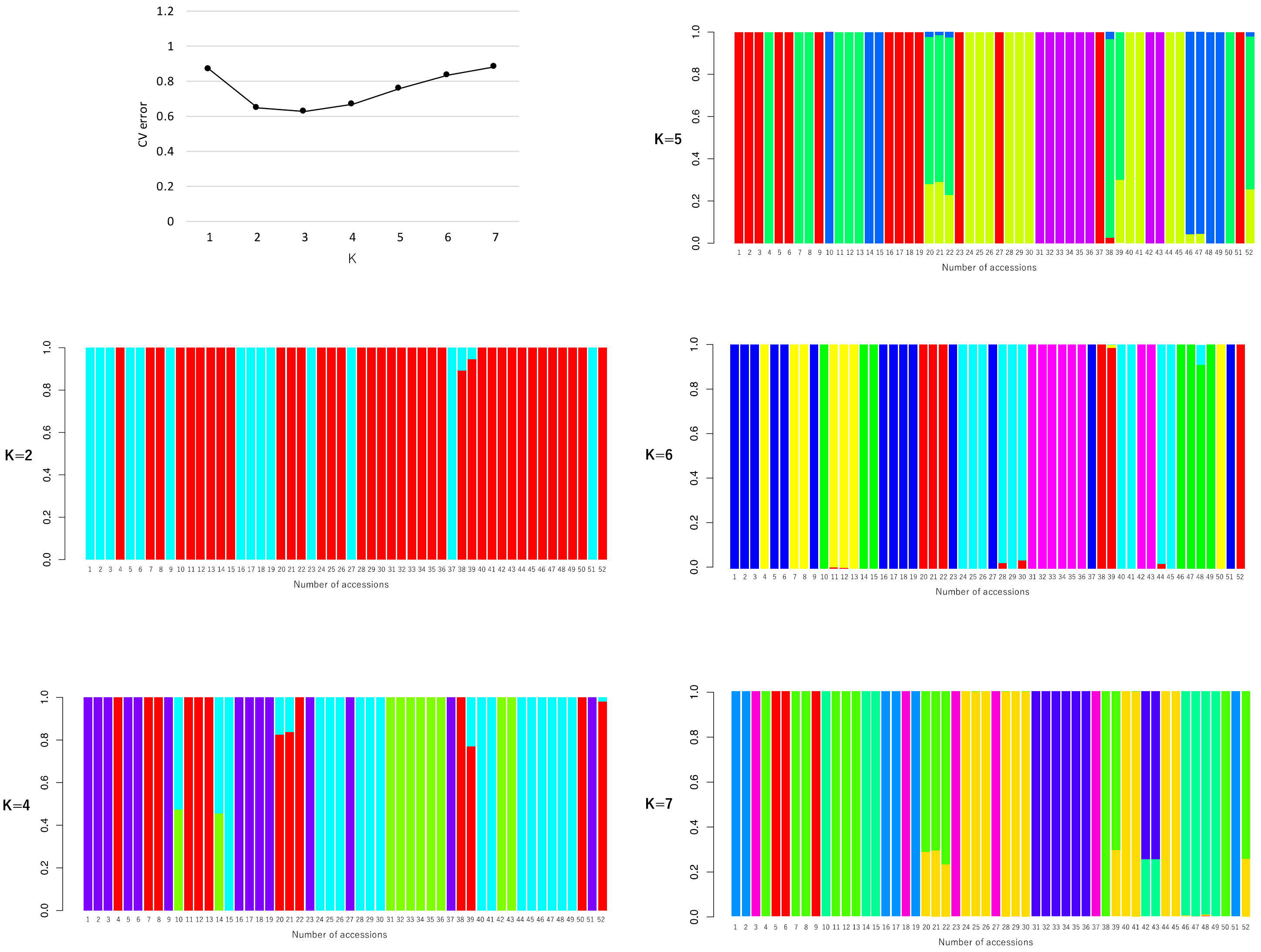

Supplementary Fig. S3. The graph of K values vs. cross-validation (CV) errors (upper left) and admixture analysis of the accessions used in this study (K=2, 4, 5, 6, 7).

Supplementary Table S1. Number of quality-filtered reads.

Number of accessions	Number of quality-filtered reads used to bowtie mapping
1	3,695,364
2	5,031,735
3	3,498,630
4	1,569,911
5	3,429,676
6	3,505,362
7	3,282,578
8	1,101,289
9	5,209,526
10	3,865,806
11	5,648,377
12	6,178,234
13	2,932,246
14	6,109,878
15	2,782,266
16	1,158,689
17	1,056,986
18	1,232,928
19	1,236,331
20	3,873,219
21	1,865,614
22	3,603,084
23	2,740,686
24	4,497,067
25	4,281,455
26	2,685,635
27	1,417,359
28	3,284,777
29	5,925,961
30	3,861,543
31	1,880,092
32	1,304,702
33	1,955,729
34	5,288,382
35	3,969,945
36	5,853,334
37	3,221,471
38	906,405
39	4,334,041
40	1,967,414
41	3,169,973
42	6,416,035
43	4,945,478
44	4,732,321
45	6,398,677
46	5,607,225
47	6,944,119
48	3,706,442
49	6,606,432
50	1,456,069
51	1,234,739
52	4,627,225

Supplementary Table S2. Pairwise F_{st} values among *L. cruciata* accessions belonging to each three populations in Japan: (a) East-Honshu, (b) West-Honshu, and (c) Kyushu group. The plotted graph of these calculated values is shown in Fig. 6.

a. East-Honshu group

	1	2	3	5	6	9	16	17	18	19	23	27	37
1		0.622	0.681	0.766	0.752	0.643	0.684	0.699	0.693	0.681	0.767	0.694	0.660
2			0.656	0.746	0.744	0.629	0.663	0.681	0.681	0.673	0.749	0.674	0.643
3				0.803	0.794	0.685	0.732	0.747	0.733	0.734	0.817	0.742	0.709
5					0.696	0.768	0.805	0.818	0.811	0.808	0.780	0.806	0.770
6						0.762	0.806	0.827	0.821	0.810	0.781	0.814	0.773
9							0.695	0.716	0.708	0.695	0.775	0.716	0.669
16								0.721	0.710	0.715	0.811	0.718	0.704
17									0.725	0.711	0.814	0.735	0.717
18										0.720	0.818	0.723	0.713
19											0.810	0.711	0.700
23												0.811	0.780
27													0.706

b. West-Honshu group

	4	7	8	11	12	13	15	20	21	22	24	25	26	28	29	30	38	39	40	41	44	45	46	47	48	49
4		0.649	0.703	0.794	0.756	0.825	0.862	0.865	0.849	0.860	0.782	0.788	0.827	0.865	0.864	0.879	0.884	0.784	0.855	0.811	0.856	0.838	0.890	0.857	0.900	0.884
7			0.699	0.784	0.743	0.813	0.854	0.853	0.838	0.856	0.776	0.779	0.819	0.855	0.855	0.866	0.877	0.775	0.846	0.802	0.848	0.829	0.880	0.850	0.887	0.868
8				0.822	0.782	0.857	0.872	0.883	0.869	0.882	0.797	0.800	0.838	0.882	0.881	0.893	0.901	0.801	0.872	0.820	0.876	0.850	0.908	0.871	0.912	0.898
11					0.704	0.778	0.848	0.825	0.822	0.832	0.766	0.768	0.808	0.838	0.838	0.851	0.873	0.769	0.834	0.793	0.835	0.813	0.860	0.833	0.864	0.853
12						0.736	0.826	0.800	0.796	0.798	0.742	0.745	0.783	0.812	0.814	0.825	0.828	0.736	0.810	0.770	0.805	0.789	0.838	0.810	0.846	0.834
13							0.862	0.845	0.830	0.843	0.776	0.785	0.819	0.852	0.854	0.870	0.879	0.782	0.847	0.804	0.852	0.824	0.878	0.846	0.876	0.864
15								0.857	0.848	0.856	0.745	0.752	0.786	0.829	0.830	0.845	0.887	0.799	0.812	0.766	0.827	0.806	0.833	0.786	0.844	0.820
20									0.827	0.832	0.760	0.763	0.807	0.841	0.834	0.849	0.878	0.778	0.833	0.797	0.834	0.817	0.868	0.832	0.873	0.858
21										0.829	0.753	0.755	0.798	0.826	0.836	0.841	0.873	0.773	0.825	0.780	0.817	0.808	0.853	0.825	0.861	0.854
22											0.763	0.766	0.805	0.837	0.841	0.848	0.887	0.779	0.836	0.787	0.829	0.813	0.863	0.830	0.869	0.856
24												0.650	0.681	0.718	0.720	0.727	0.797	0.715	0.711	0.675	0.710	0.696	0.758	0.726	0.749	0.750
25													0.686	0.723	0.723	0.732	0.802	0.716	0.708	0.676	0.716	0.697	0.759	0.732	0.765	0.749
26														0.767	0.767	0.775	0.836	0.752	0.759	0.713	0.761	0.741	0.804	0.765	0.796	0.791
28															0.791	0.805	0.877	0.783	0.796	0.748	0.788	0.758	0.841	0.800	0.844	0.828
29																0.803	0.877	0.781	0.800	0.755	0.776	0.763	0.834	0.808	0.842	0.823
30																	0.891	0.791	0.811	0.760	0.790	0.770	0.840	0.813	0.851	0.837
38																		0.794	0.861	0.830	0.863	0.861	0.898	0.868	0.902	0.888
39																			0.775	0.743	0.771	0.761	0.805	0.783	0.817	0.801
40																				0.733	0.791	0.772	0.820	0.786	0.815	0.817
41																					0.741	0.729	0.781	0.748	0.784	0.770
44																						0.748	0.832	0.796	0.820	0.815
45																							0.809	0.780	0.812	0.802
46																								0.801	0.842	0.826
47																									0.805	0.791
48																										0.834

c. Kyushu group

	10	14	31	32	33	34	35	36	42	43
10		0.768	0.871	0.877	0.873	0.852	0.854	0.852	0.825	0.829
14			0.858	0.864	0.861	0.847	0.851	0.849	0.814	0.814
31				0.667	0.616	0.755	0.773	0.770	0.867	0.865
32					0.666	0.773	0.794	0.781	0.876	0.866
33						0.751	0.786	0.781	0.872	0.866
34							0.723	0.710	0.837	0.842
35								0.739	0.835	0.838
36									0.833	0.838
42										0.806

Supplementary Table S3. List of 77 kinds of SNP sites causing mutations in protein amino acid sequences between East- and West- Japan populations.

Scaffold	Scaffold position (bp)	East	West	Mutation	Amino acid size	Protein name（only annotated）
scaffold185_cov145	10218709	A	G	Y8C	/37	
scaffold14169_cov154	465311	T	C	Y68H	/86	
scaffold391_cov159	16834	A	T	Y40F	/721	PREDICTED: long-chain fatty acid transport protein 4 isoform X1 [Tribolium castaneum]
scaffold265_cov151	961484	T	C	Y331H	/1828	
scaffold437_cov154	291326	T	C	Y1846H	/2668	
scaffold7_cov146	7755545	G	A	V7M	/40	
scaffold83005_cov147	242285	G	A	V60I	/172	
scaffold21_cov148	11506702	G	A	V51I	/82	
scaffold14268_cov142	141803	T	C,	V35A	/1375	PREDICTED: uncharacterized protein LOC103313681 [Tribolium castaneum]
scaffold83012_cov144	9280976	C	G	T535S	/1021	
scaffold331_cov144	12605319	C	G	T502S	/950	
scaffold7_cov146	10823816	A	G	T20A	/42	
scaffold431_cov152	1229751	A	T	T113S	/182	
scaffold65_cov146	373462	A	G	T1011A	/1161	PREDICTED: uncharacterized protein LOC107397770 isoform X1 [Tribolium castaneum]
scaffold9_cov147	37014487	A	G	T1006A	/2222	
scaffold192_cov135	6445929	C	T	S778L	/2102	PREDICTED: valine--tRNA ligase [Tribolium castaneum]
scaffold353_cov126	1542083	T	C	S70P	/260	activating transcription factor of chaperone [Tribolium castaneum]
scaffold44_cov143	2972197	T	A	S657T	/1581	
scaffold391_cov159	16844	T	C	S37P	/1442	PREDICTED: long-chain fatty acid transport protein 4 isoform X1 [Tribolium castaneum]
scaffold313_cov148	1400182	C	G	S27W	/124	
scaffold83013_cov142	14097215	T	A,T	S23T	/199	
scaffold39_cov144	11518871	T	A	S16T	/225	
scaffold83014_cov135	70215	A	G	S133G	/309	
scaffold83005_cov147	1839884	C	T	R57C	/61	
scaffold104_cov151	11944293	G	A	R40K	/164	PREDICTED: myosin light chain alkali [Tribolium castaneum]
scaffold185_cov145	10218721	C	T	P4L	/74	
scaffold13571_cov146	7562321	C	G	P432R	/714	PREDICTED: neprilysin-11 isoform X2 [Tribolium castaneum]
scaffold192_cov135	6413578	C	A	P33T	/229	PREDICTED: transcription initiation factor TFIID subunit 9 [Tribolium castaneum]
scaffold63_cov143	4512815	C	G	P145R	/303	
scaffold21_cov148	19421939	C	T	P1259S	/1621	
scaffold101_cov148	2225981	A	G	N51D	/82	
scaffold101_cov148	1244801	C	G	N452K	/467	PREDICTED: UDP-glucose 6-dehydrogenase isoform X1 [Tribolium castaneum]
scaffold150_cov151	3302897	A	G	N44S	/119	
scaffold313_cov148	1400172	C	G	N30K	/62	
scaffold192_cov135	5861355	A	G	N26S	/119	
scaffold30_cov139	12949237	A	G	M349V	/2362	
scaffold83014_cov135	1621760	A	G	M286V	/313	
scaffold179_cov142	88895	C	G	L91V	/129	PREDICTED: histone H3.3-like [Dinoponera quadriceps]
scaffold33_cov153	4057601	T	A,T	L50H	/70	
scaffold142_cov138	39558	A	T	L4F	/342	cathepsin L precursor [Tribolium castaneum]
scaffold185_cov145	4942431	C	T	L46F	/73	
scaffold39_cov144	15346364	C	A,C	L28I	/2324	
scaffold104_cov151	11466361	A	T	L196F	/331	PREDICTED: venom acid phosphatase Acph-1 [Tribolium castaneum]
scaffold391_cov159	239559	T	G	L140V	/350	PREDICTED: protein catecholamines up [Tribolium castaneum]
scaffold83010_cov147	319644	A	T	K56*	/56	
scaffold79_cov139	4758675	G	C	K41N	/96	
scaffold192_cov135	7391120	A	G	K33E	/805	PREDICTED: putative mediator of RNA polymerase II transcription subunit 26 isoform X3 [Tribolium castaneum]
scaffold9_cov147	34160787	A	G	K320E	/1448	
scaffold185_cov145	10393102	A	T	K128N	/445	PREDICTED: protein odr-4 homolog [Tribolium castaneum]
scaffold63_cov143	1259504	A	G	K11R	/412	nicotinic acetylcholine receptor alpha 9 subunit precursor [Tribolium castaneum]
scaffold79_cov139	2543208	T	G	I73S	/141	
scaffold60_cov140	448409	A	G	I71M	/509	PREDICTED: putative oxidoreductase GLYR1 homolog [Apis mellifera]
scaffold44_cov143	2972198	T	C	I657T	/3162	
scaffold14169_cov154	1034367	A	G	I5M	/39	
scaffold101_cov148	2214485	T	C	I52T	/196	
scaffold34_cov149	5150000	A	G	I417M	/855	PREDICTED: ubiquitin-protein ligase E3A [Tribolium castaneum]
scaffold681_cov82	2380366	A	G	I38M	/91	
scaffold39_cov144	10387316	T	C,	I30T	/43	
scaffold170_cov139	5112645	T	G	I30S	/35	
scaffold14169_cov154	496211	T	C	I21T	/1531	PREDICTED: uncharacterized protein LOC664116 isoform X1 [Tribolium castaneum]
scaffold63_cov143	2616416	T	C	I200T	/540	
scaffold83013_cov142	4630861	T	C	I1542T	/1683	PREDICTED: LOW QUALITY PROTEIN: RING finger protein 17 [Bombus terrestris]
scaffold100_cov139	3574246	A	G	I123V	/258	
scaffold6_cov141	5926622	T	C	I111T	/434	yellow-3 [Tribolium castaneum]
scaffold21_cov148	12750837	A	G	H53R	/53	
scaffold83013_cov142	20193649	G	C	G48R	/77	
scaffold83015_cov127	2817043	G	T,G	G14*	/194	PREDICTED: EF-hand domain-containing protein D2 homolog [Tribolium castaneum]
scaffold101_cov148	752499	T	A,G	F174L	/506	PREDICTED: rhythmically expressed gene 2 protein [Tribolium castaneum]
scaffold80_cov132	924828	A	G	E65G	/72	
scaffold13571_cov146	123010	T	G	D136E	/311	
scaffold179_cov142	2095631	G	T	C371F	/489	PREDICTED: GA-binding protein subunit beta-1 isoform X2 [Tribolium castaneum]
scaffold20_cov145	5035306	C	G	A86G	/370	PREDICTED: hydroxylysine kinase [Tribolium castaneum]
scaffold313_cov148	11873679	C	T	A191V	/265	PREDICTED: protein SCO1 homolog, mitochondrial [Tribolium castaneum]
scaffold118_cov151	19262293	G	A	A154T	/360	PREDICTED: galectin-8 isoform X1 [Tribolium castaneum]
scaffold83010_cov147	4691449	C	T	A1047V	/1337	PREDICTED: nidogen-1 [Tribolium castaneum]
scaffold43_cov144	232379	A	A,G	*1609W	/4310	PREDICTED: bromodomain adjacent to zinc finger domain protein 2B isoform X10 [Tribolium castaneum]
scaffold277_cov148	7848449	A	G	*1397W	/2135	PREDICTED: sortilin-related receptor [Tribolium castaneum]
